# The Feasibility of a Beating-Heart Transplant From Brain-Dead Donors

**DOI:** 10.3389/ti.2025.13921

**Published:** 2025-04-11

**Authors:** Igor Vendramin, Sandro Sponga, Alessandro Di Lorenzo, Sandro Nalon, Uberto Bortolotti, Ugolino Livi, Andrea Lechiancole

**Affiliations:** ^1^ Department of Medicine, University of Udine, Udine, Italy; ^2^ Cardiothoracic Department, University Hospital, Udine, Italy

**Keywords:** graft preservation, heart transplantation, organ care system, beating heart, donor after brain death

Dear Editors,

Prolonged donor graft ischemia during retrieval and transportation may be responsible for allograft dysfunction after heart transplantation (HTx) [1]. Therefore, limiting the ischemic period and the deleterious effects of ischemia-reperfusion on the donor graft may have a favorable impact on the outcome of HTx; this would also allow longer distance procurements and acceptance of marginal grafts. This has been demonstrated using special graft preservation modalities such as those obtained when employing the Organ Care System (OCS^TM^; *TransMedics Inc., Andover, MA, USA*) [2, 3].

Using beating grafts from donors after brainstem death (DBD) further reduces the ischemic periods in HTx by avoiding a second cardioplegic arrest; this is of the utmost importance in HTx with donors after circulatory death (DCD). This has been achieved in two patients and is described in the following report, an experience which may be considered a prelude to an ischemia-free HTx.

Both recipients gave their informed consent and the Institutional Review Board approved the procedures. Most of the relevant data of the recipients and donors are summarized in Table 1.

**TABLE 1:** Donor and recipient data.

	Recipient 1	Recipient 2
Age (years), sex	69, male	63, male
Indication for HTx	Post-op LV failure	Ischemic CM
Pre-HTx status	Impella, VA-ECMO	IABP
Donor age (years), sex	51, male	63, male
List priority	Emergent	Urgent
Time on OCS (minutes)	256	229
Total ischemic time (minutes)	35	47
Cardiopulmonary bypass (minutes)	177	161
Recipient aorta cross clamp (minutes)	88	71
Post-HTx course	Moderate rejection, resolved	Uncomplicated

The donor hearts were arrested with cold antegrade cardioplegia, and the longest possible segment of ascending aorta was retrieved during cardiectomy. Hearts were placed on OCS after 35 and 47 min of ischemia, respectively, and then the graft was perfused with warm oxygenated donor blood through an aortic line and vented through a pulmonary arterial line. Technical details of OCS implant in our center have been previously described [3]. During transportation, constant monitoring of heart rate, aortic pressure, coronary flow, and lactate profile revealed no anomalies.

Prior to donor heart arrival, the recipients were prepared and placed on a cardiopulmonary bypass (CPB), the left ventricles were vented through the right superior pulmonary vein, and cardiectomy was carried out. Once the donor grafts reached the operative room the setup was modified so that they could be perfused through the CPB circuit. An additional arterial perfusion line, long enough to reach the OCS device, was connected, under sterile conditions, to the CPB circuit (Figure 1A). After the aortic cannula connector was loosened, the donor heart was manually rotated 180°, exposing its anterior aspect. The donor aorta was cannulated near the aortic sinotubular junction with a cardioplegia needle and connected to an additional arterial line of the recipient CPB circuit (Figure 1B). A bolus of 1g of metilprednisolone was administered and the donor heart rate was maintained at 80 beats/minute through ventricular bipolar pacing wires. Perfusion from the OCS was stopped, the aorta was cross-clamped immediately distal to the CBP perfusion line, and antegrade flow was initiated from the CPB circuit with a separate roller pump through the cardioplegia needle (Figure 2A); a target flow of 300–500 cc/min and a pressure of about 250–300 mmHg to achieve an aortic root pressure of 100 mmHg were maintained [4]. While moving the graft into the operative field, collection of blood loss was assured by placing the heart into a basin.

**FIGURE 1:**
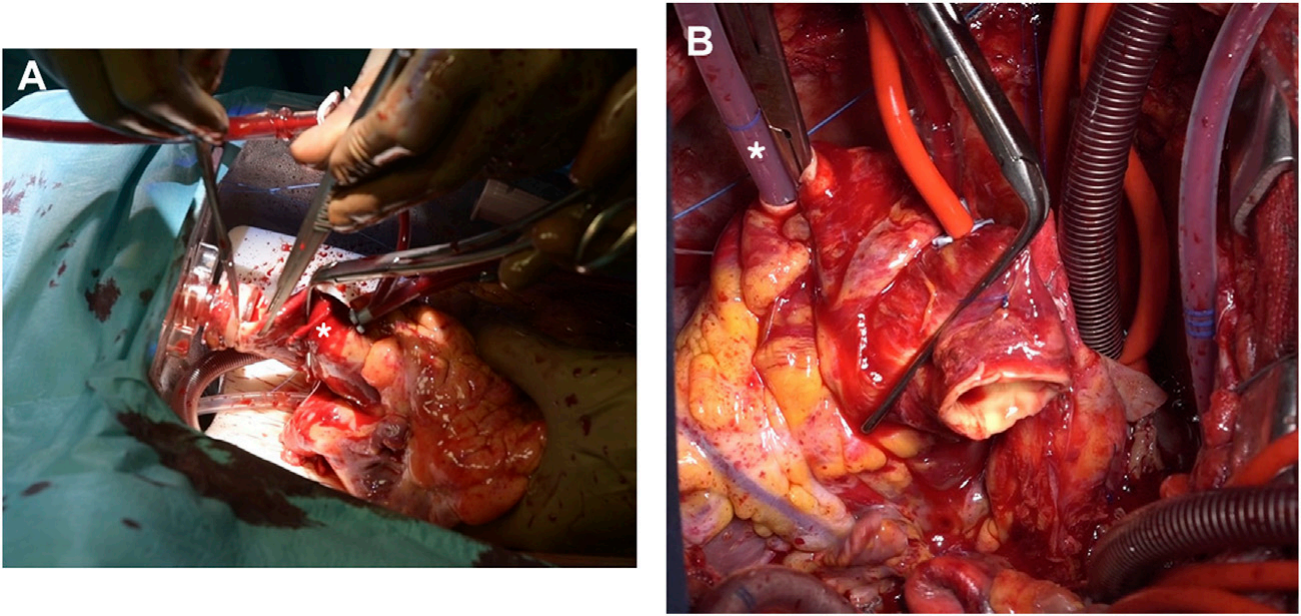
**(A)** The OCS platform is kept close to the operative table and a separate arterial perfusion line (arrow), long enough to reach the donor graft, is primed. **(B)** A standard cardioplegia cannula is inserted proximally in the donor aorta (arrow) and connected to the perfusion line (asterisk).

**FIGURE 2:**
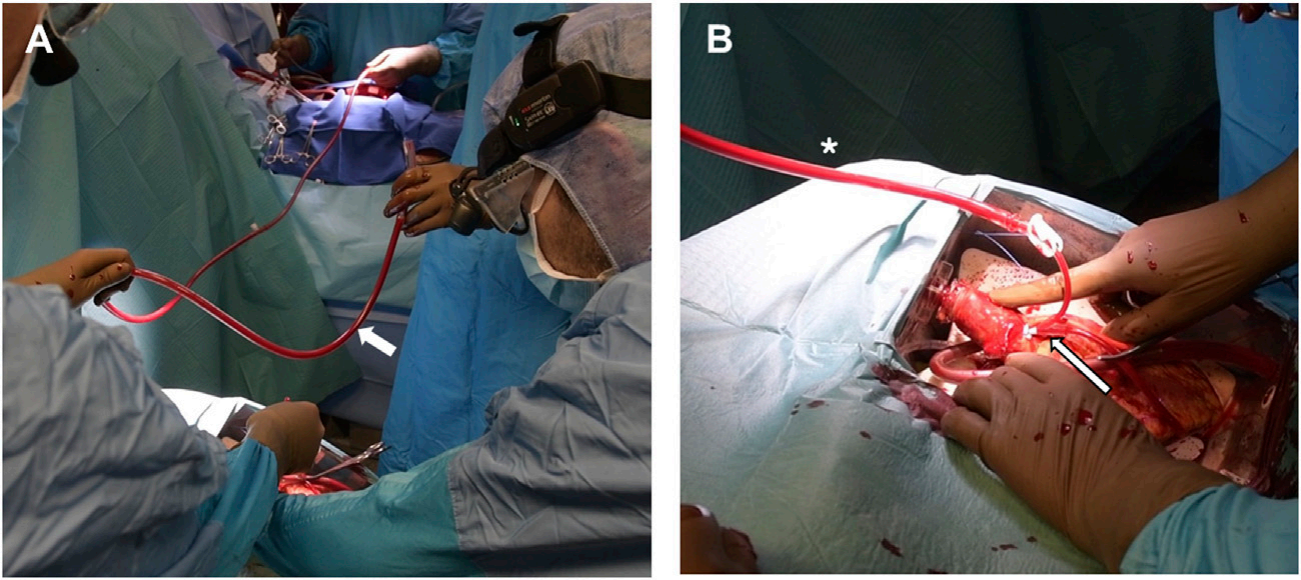
**(A)** The donor aorta is cross-clamped (asterisk) and the donor graft is detached from the OCS by cutting its distal ascending aorta immediately under the connector. **(B)** During implant, the OCS pulmonary cannula is removed and replaced by a smaller and more pliable tube to vent the right ventricle (asterisk).

Graft implantation started with the left atrial anastomosis. Of paramount importance during this phase was avoiding any twisting or kinking of the perfusion line and of the graft aorta, which was frequently palpated to verify adequate pressure was maintained. The left ventricular vent was left in place while sewing the left atrial cuff and the OCS pulmonary artery cannula was replaced with a soft vent positioned inside the right ventricle and secured through a purse-string suture to the pulmonary artery (Figure 2B). The aortic anastomosis was then carried out between the two clamps, which were released upon completion of the suture. The remainder of the operation was concluded, as in a standard HTx, by sequentially anastomosing the pulmonary arteries and the inferior and superior vena cava.

Procedural details are reported in Table 1. At the end of the HTx, Recipient 1 required moderate inotropic support with epinephrine, while Recipient 2 was weaned from CPB without difficulty. Recipient 1 presented moderate rejection after two days, which resolved after treatment. In Recipient 2, the postoperative course was uncomplicated.

In cases where prolonged ischemic times are required for donor graft procurement, myocardial stunning leads to a higher need for mechanical and inotropic support post-HTx [5]. Therefore, limiting warm and cold ischemic periods is a prerequisite to minimize myocardial injury in the donor heart and potentially achieve a smoother postoperative course.

The conventional static cold storage prevents safe graft preservation when long-distance procurement with extended ischemia times is required [6]. For this reason, we recently shifted to the OCS technique as the preferred method for donor graft protection during transportation; with this technique, we have obtained promising results when HTx was performed in high-risk recipients employing donor marginal grafts or in patients bridged to HTx on mechanical support [2, 3]. The lesson learned from this favorable experience indicates that, when possible, greater reduction of ischemic times or even an ischemia-free procedure should be aimed for in HTx.

With this in mind, we have recently performed two HTx using beating grafts from DBD with the advantage of eliminating the initial period of warm ischemia when using a graft from DCD [4].

This preliminary experience demonstrates that HTx with a beating heart obtained by DBD is feasible, and the brief warm ischemia required to institute OCS support does not adversely influence patient outcome. However, it is more cumbersome compared to a standard HTx, due to difficulty in manipulating a moving graft while performing the left atrial anastomosis; there are also concerns regarding longer operative times and additional costs. In fact, compared to traditional ice-cold storage, the OCS platform is rather expensive and requires a trained staff able to manage *ex-situ* perfusion and possible device troubles. Therefore, the widespread use of this procedure may be limited by financial and logistical reasons.

Nevertheless, although eliminating even a brief period of ischemia might not significantly influence HTx results in low-risk recipients, the surgical complexity should be counterbalanced by beneficial effects, especially when using marginal donors, DCD, or long-distance procurements. These expectations will have to be confirmed by studies conducted on larger populations.

With this technique, only one cardioplegia infusion is required, but retrieving the beating donor heart without any cardioplegic arrest, as recently reported in a DBD [5], may provide additional benefit by avoiding any ischemia-reperfusion injury, ideally leading to an ischemia-free HTx in all cases.

## Data Availability

The raw data supporting the conclusions of this article will be made available by the authors, without undue reservation.

## Abbreviations

DBD, Donation after Brainstem Death; DCD, Donor after circulatory death; HTx, Heart transplantation; OCS, Organ Care System; CPB, Cardiopulmonary bypass; AV ECMO, Artero-venous extracorporeal membrane oxygenation.
